# C_60_ Intercalated Graphite as Nanolubricants

**DOI:** 10.3390/ma3094510

**Published:** 2010-08-27

**Authors:** Kouji Miura, Makoto Ishikawa

**Affiliations:** Department of Physics, Aichi University of Education, Hirosawa 1, Igayacho, Kariya 448-8542, Japan; E-Mail: makoishi@auecc.aichi-edu.ac.jp

**Keywords:** C_60_ fullarene, graphene, graphite, intercalate, lubricant, superlubricity

## Abstract

We synthesized the novel nanocomposite consisting of alternately stacked single graphene sheets and a C_60_ monolayer by using the graphite intercalation technique in which alkylamine molecules help intercalate large C_60_ molecules into the graphite. It is found that the intercalated C_60_ molecules can rotate in between single graphene sheets by using ^13^C NMR measurements. The grease with the nanocomposite materials provides a much better lubricating performance than that with other additives that have been well-known up to now. This result exhibits that a C_60_ monolayer intercalated between graphenes plays an important role in lubricating behavior.

## 1. Introduction

Nanocomposites composed of an organic polymer and inorganic layered host are a new type of composite that have been developed recently, and have unique properties. Since van der Waals forces dominantly acting between successive layers of graphite are relatively weak, it is possible for a wide range of atoms, molecules, and ions to intercalate between graphite layers, thus producing the graphite intercalation compounds (GICs) [1]. However, it is difficult to intercalate organic molecules or polymers directly into the interlayer of graphite through an ion exchange reaction to obtain the polymer/graphite nanocomposites, particularly large molecules. Here we show nanocomposite materials consisting of alternately stacked single graphene sheets and C_60_ monolayers, in which the C_60_ molecules can rotate in between single graphene sheets. We synthesized this novel nanocomposite by the graphite intercalation technique in which alkylamine molecules help intercalate large C_60_ molecules into the graphite. This provides a general preparation method for intercalating huge fullerene molecules into graphite, which will lead to promising materials with novel mechanical, physical, and electrical properties.

## 2. Experimental

First, graphite oxide (GO) was synthesized from graphite powder with an average size of 500 μm (Nippon Carbon Co., Ltd.) in accordance with the Hummers method [1] with some modifications, and the intercalation of octylamine into graphite (GO-OA) was performed as described previously [2,3]. Next, octylamine-intercalated graphite oxide (GO-OA) was added to fullerene solution (100 mg of C_60_ dissolved in 100 ml toluene), and after that, the toluene used was evaporated at room temperature, leaving behind in the products (GO-OA-C_60_). The C_60_ fullerene used in this experiment was purchased from Frontier Carbon Co., Ltd, in Japan. The products of GO-OA-C_60_ were treated with 0.1 N hydrochloric acid solution at room temperature for at least 30 minutes and dried in air at 80 °C overnight to remove the octylamine, resulting in the C_60_-intercalated graphite oxide (GO-C_60_). Finally, in order to remove C_60_ powders that are not intercalated into the graphite, and moreover, to remove octylamines that are intercalated into the graphite, GO-C_60_ was heated for at least 80 minutes at 600 °C under a high vacuum of 10^−6^ Torr, which results in the nanocomposite consisting alternately of a stacked single graphene sheet and a C_60_ monolayer. All specimens were analyzed by using X-ray diffraction (Rigaku RINT 2200/PC diffractometer: CuKα radiation at 40 kV and 30 mA), FT-IR spectroscopy (FTIR：JASCO 480 Plus FT-IR spectrometer: the samples in KBr pellets), NMR (our original 7.1 T spectrometer with a Tecmag Apollo spectrometer and a Doty SuperSonic MAS 7 mm probe head).

## 3. Results and Discussion

**Figure 1:**
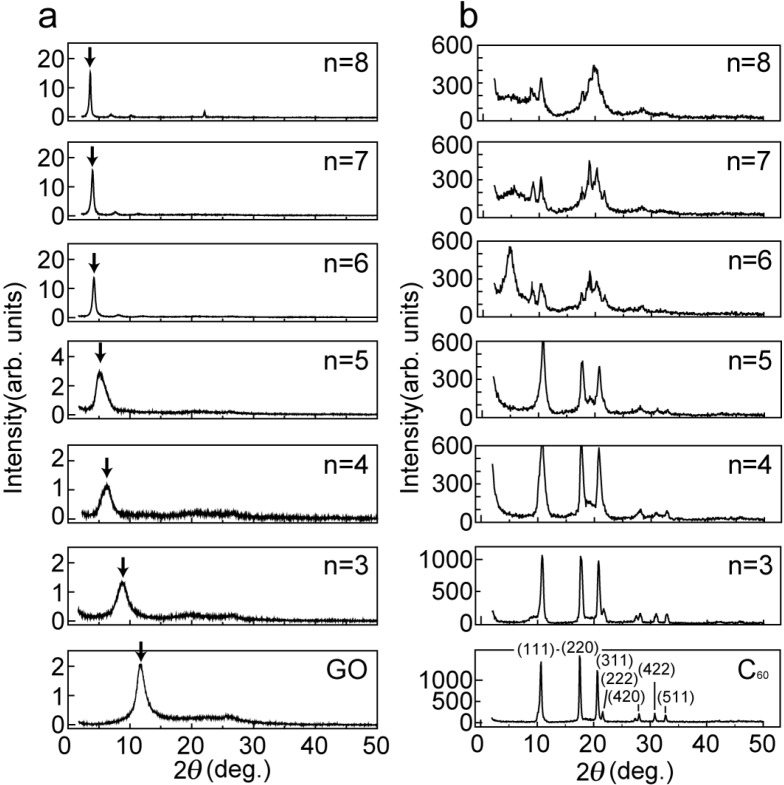
XRD intensity from as-prepared samples. (**a**) XRD intensity from graphite oxide (GO) and alkylamine-intercalated graphite oxides (GO-amine) with different chain lengths (C_n_H_2n+1_NH_2_) (n = 3–8). The peaks of GO and GO-amine are indicated by the arrows in the figure; (**b**) XRD intensity from GO-amine with different chain lengths in C_60_ solution (n = 3–8).

**Figure 2:**
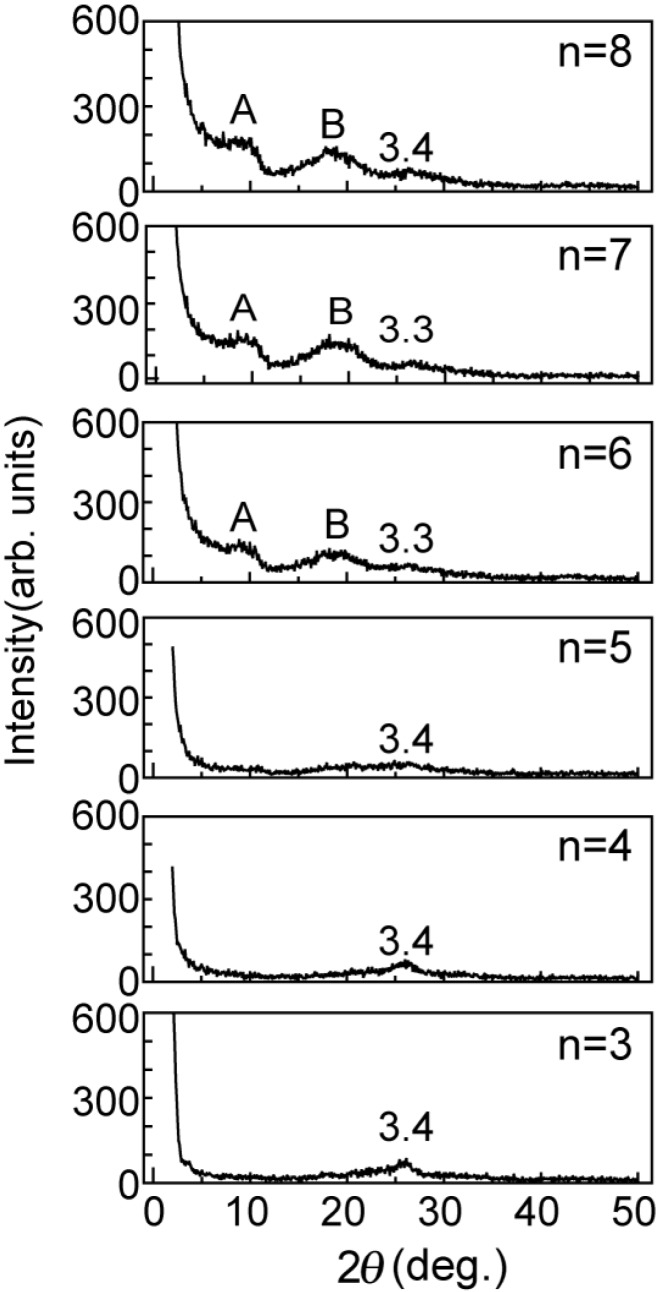
XRD intensity from the GO-C_60_ specimens of Figure 1b after heating for 80 minutes at 600 °C under a high vacuum of 10^−6^ Torr.

Figure 1a shows the X-ray diffraction (XRD) intensity of graphite oxide (GO) and alkylamine-intercalated graphite oxide (GO-amine) with different alkyl chain lengths (C_n_H_2n+1_NH_2_) (n = 3 to 8). The appearance of a peak in the GO sample of Figure 1a shows that the spacing between graphite oxide sheets is approximately 8 Å, which is identical to the published data [1]. It was found that the spacing between graphite oxide sheets in the case of the GO-amine (n = 3 to 8) increases with the increase in the length of the alkyl chain incorporated in the interlayer space of the GO. Figure 1b shows the XRD intensity of the GO-amine (n = 3 to 8) in C_60_ solution, which we call the C_60_-intercalated graphite oxide (GO-C_60_). It should be noted that there appear drastic changes in the XRD intensity between n = 5 and n = 6 in Figure 1b, which indicates that C_60_ molecules are intercalated in the interlayer space of the GO by the driving force of alkylamine situated between the graphene oxide sheets when the interlayer space is sufficiently larger than the C_60_ molecule. However, there exist many C_60_ powders which are not intercalated into the GO in these GO-C_60_ specimens, because the stronger XRD intensity peaks, (111), (220), (311), (222), (331), (420), (422) and (511), from C_60_ powders (JCPDS file No.44-0558) appear in the spectra of Figure 1b. In order to remove C_60_ powders that are not intercalated into the GO, and moreover, to expel the alkylamines that are intercalated into the graphite, the GO-C_60_ specimens shown in Figure 1b were heated for 80 minutes at 600 °C under a high vacuum of 10^−6^ Torr, as shown in Figure 2a.

**Figure 3:**
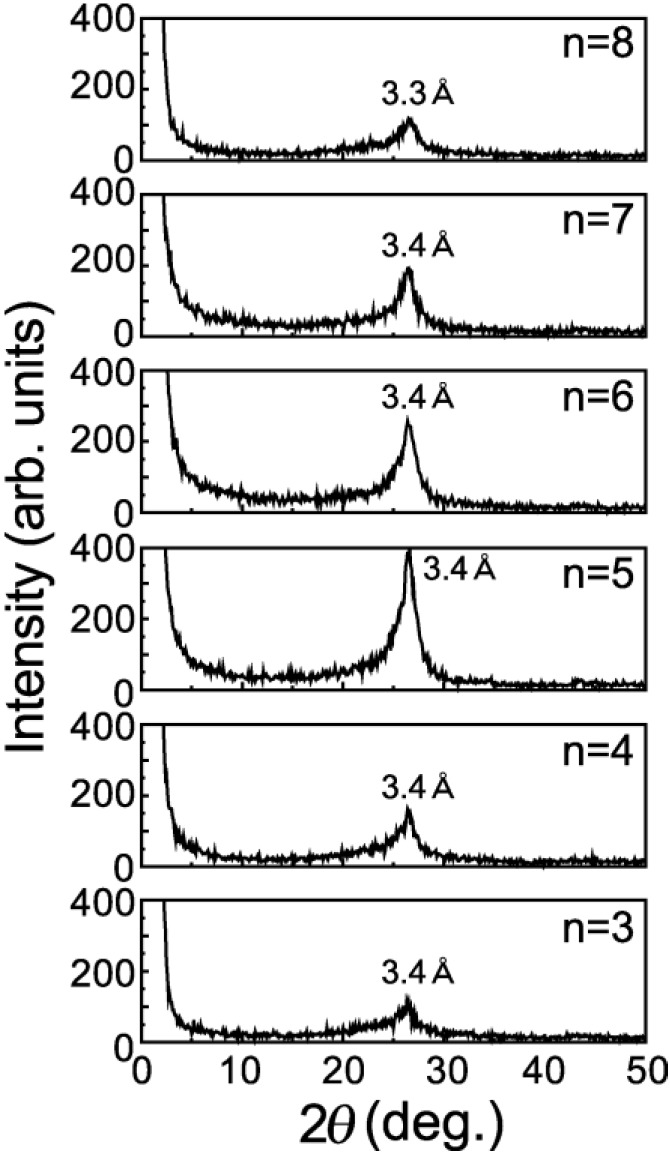
XRD intensity from the alkylamine-intercalated graphite oxide (GO-amine) specimens after heating for 80 minutes at 100 ºC under a high vacuum of 10^−6^ Torr.

It should be noted that n = 6, n = 7 and n = 8 have broad peaks of A and B (as shown in Figure 2) corresponding to *d*-spacings of 9 Å and 4.6 Å, respectively, in addition to a broad peak of *d* = 3.3 Å corresponding to the spacing of graphite layers. n = 3, n = 4 and n = 5 have only a single broad peak of *d* = 3.3 Å. Since the GO-amine reverts to the graphite layers when alkylamines leave the GO-amine host after heating at up to 100 °C, as shown in the XRD intensity of Figure 3, the graphene oxide layers in the GO-C_60_, which do not include C_60_ molecules, also revert to the graphite layers when alkylamines go out from it after heating. It is expected that the peaks of A and B (n = 6 to 8) in Figure 2 are due to the *d*-spacing between the graphenes intercalating the C_60_ monolayer and their stacking faults, respectively. However, since peak A is also widely distributed, the *d*-spacings between the graphenes intercalating the C_60_ monolayer are considered to be widely distributed.

**Figure 4:**
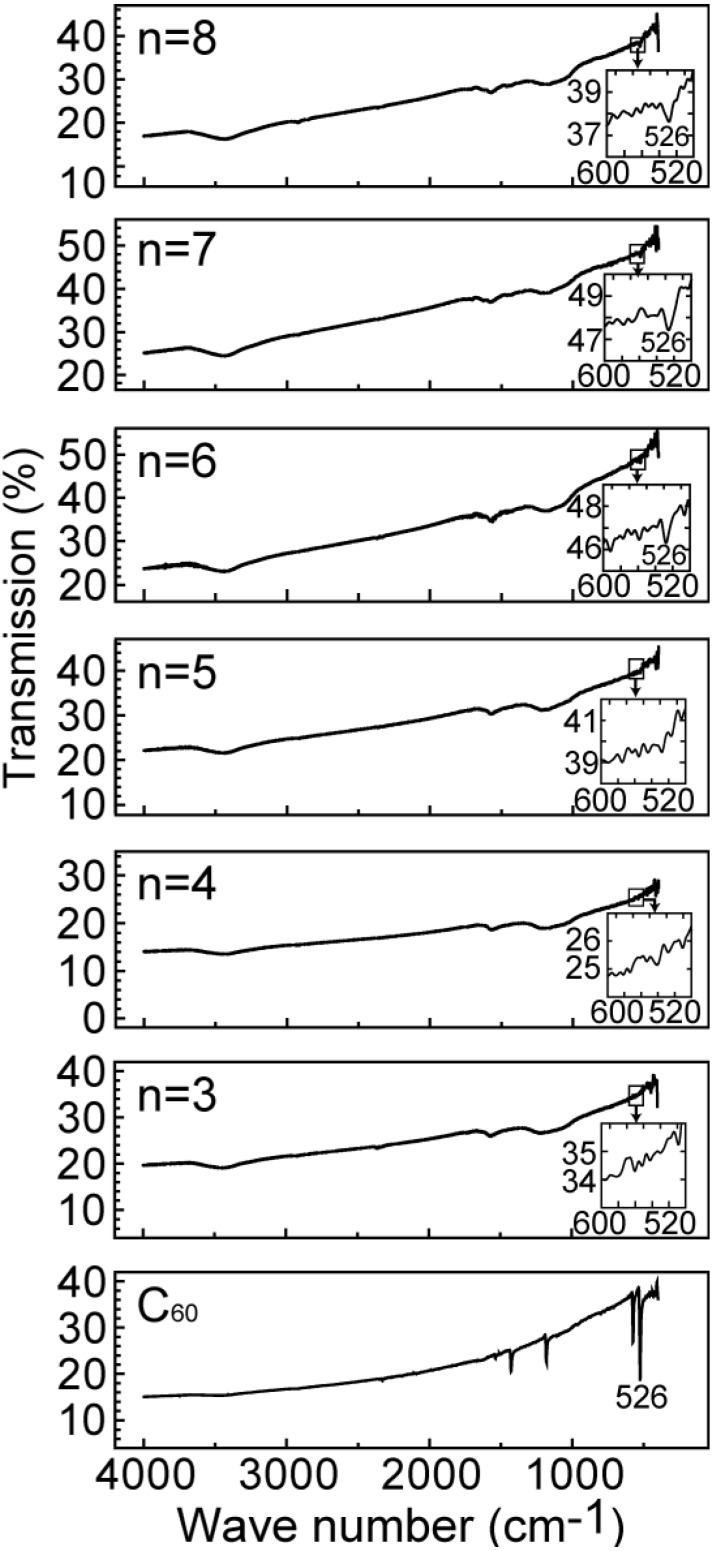
FT-IR spectra for the specimens of Figure 2. The C_60_ intermolecular IR-active (*F*_1u_) modes are indicated by the inset of each panel of the figure.

Figure 4 show the FT-IR spectra for the specimens of Figure 2. The FT-IR spectra from n = 6, n = 7 and n = 8 in Figure 2b exhibit the C_60_ intermolecular IR-active (*F*_1u_) modes [4], although those with the shorter alkylamine chains do not exhibit these modes. This result is consistent with the conclusion based on Figure 2 that C_60_ molecules can be intercalated into the GO with alkylamine chains longer than that of n = 5. However, one mode of 526 cm^−1^ among IR-active (*F*_1u_) modes only appears in these FT-IR spectra because the number of C_60_ molecules included in the specimens is rather small.

**Figure 5:**
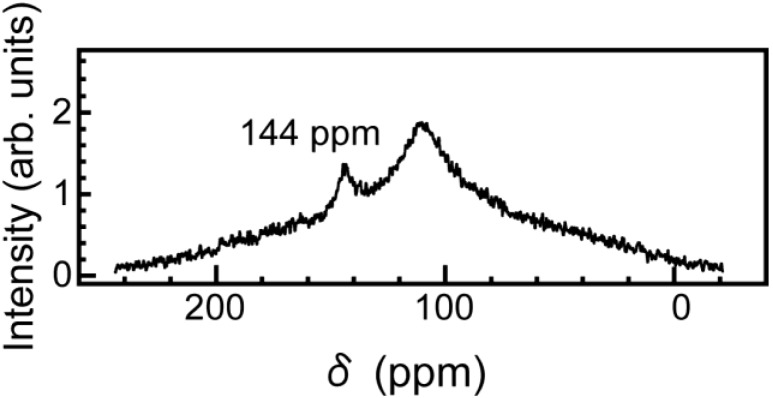
The ^13^C NMR spectra for the specimens shown in Figure 2 at room temperature. Only one sharp line with a peak position of 144 ppm [4] was observed.

The rotational dynamics of C_60_ molecules between graphenes have been investigated by ^13^C NMR in the temperature range from room temperature to −80 °C. We prepared C_60_ materials 20–30% enriched in ^13^C in order to increase the ^13^C NMR signal. The present specimen was mixed with Na_2_SO_4_ in a weight ratio of 1:50 to avoid arcing in an NMR probe. The NMR experiments were performed at 75.4 MHz for ^13^C in an external field of 7.1 T by the pulse inversion recovery method. ^13^C NMR spectra were taken by Fourier transforming the signal following the π/2 pulse. The typical π/2 pulse width was 5.4 μs. It is well known that for C_60_ molecules in solid C_60_ at room temperature, large rotational motion averages out the chemical-shift anisotropy (CSA) and the ^13^C NMR spectra show motional narrowing of 2.5 ppm in width. In contrast, spectra broaden at low temperature and develop the CSA power pattern with a CSA tensor with the principle values δ_11_ = 213 ppm, δ_22_ = 182 ppm, and δ_33_ = 33 ppm [4]. Figure 5 shows the ^13^C NMR spectra for the specimens of Figure 2 at room temperature, where the ^13^C NMR spectrum at room temperature is the same as that at the temperature of −80 °C. Only one sharp line with a peak position of 144 ppm was observed, and its line shape is a Gaussian-like function with an FWHM value of 5 ppm. The positions are in good agreement with the average principle values for C_60_ molecules in solid C_60_, and the linewidth is about one-twentieth narrower than that of the powder pattern. These observations clearly demonstrate the lack of the polymerization [5] of C_60_ molecules in the present material. Furthermore, the observed Gaussian-like line shape means a motional narrowing and that C_60_ molecules rotate quasi-freely with a correlation time on the order of 10 ps. This correction time is similar to that of the same case [6]. This means that no strong bonding such as chemical bonding between the graphenes and C_60_ molecules is made, and C_60_ molecules easily rotate for outer force.

**Figure 6:**
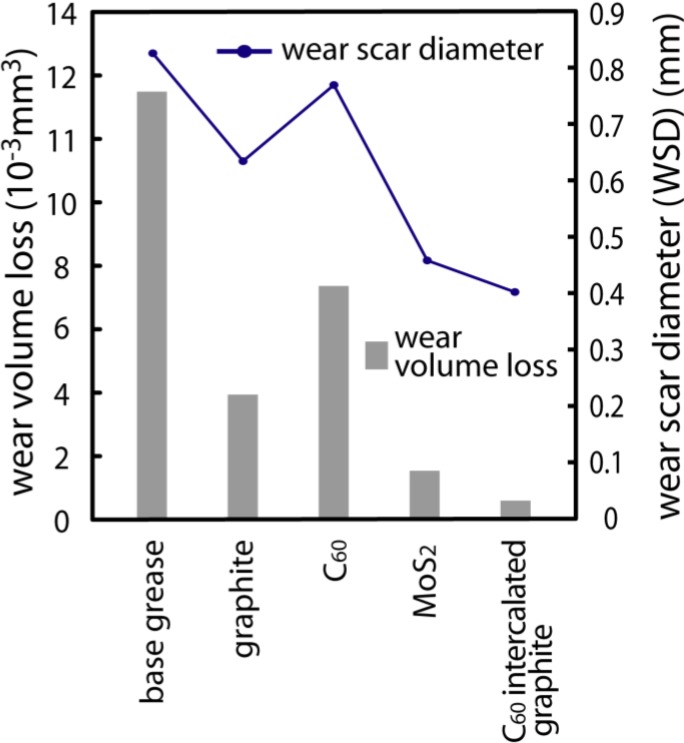
The wear scar diameter (WSD) and wear volume loss of the test ball for the grease with various kinds of additives.

Many lubricant agents contain solid particles, such as commercial colloidal dispersions of graphite, tungsten disulfide, molybdenum disulfide (MoS_2_), or polytetrafluoroethylene as additive to enhance the lubricating ability of the base oil or grease. Their lubricating properties are generally attributed to a layered structure on the molecular level with weak bonding between layers. Such layers are able to slide relative to each other with minimal applied force, thus giving them their good tribological properties. We added this nanocomposite to the commercially available grease and evaluated the performance of that as the additive of lubricant. The lubricating performance is tested using a four-ball tester with four balls. The machine including the four-ball tribosystem has been used to determine the lubricant properties. The four-ball test is the industrial standard test method for measuring the wear preventive characteristics of a lubricant. Placed in a bath of the test lubricant, three fixed steel balls are put into contact with a fourth ball in rotating contact at set conditions. Lubricant wear protection properties are measured by comparing the average wear scars on the three fixed balls. The smaller the average wear scar, the better the protection. The nanocomposite was added to the base grease and the lubricant wear protection properties of the grease were investigated. Similarly, the lubricating properties of the greases blended with widely used additives such as graphite and MoS_2_ were also studied. The lubricating tests were carried out under the lubrication of 2 g grease containing 1.0% additives. Those were evaluated by measuring the wear scar diameter (WSD) and wear volume loss of the test ball. The test was conducted at a rotating speed of 1200 rpm and a constant load of 441 N for 1 h. The results of the wear behaviors for some lubricating additives are shown in Figure 6. According to Figure 6, the WSD and wear volume loss of the test ball for the base grease were 0.83 mm and 11.7 × 10^−3^ mm^3^, respectively. The WSD and wear volume loss of MoS_2_ were 0.46 mm and 0.9 × 10^−3^ mm^3^, respectively. The WSD and wear volume loss of graphite were 0.68 mm and 2.9 × 10^−3^ mm^3^, respectively. It is found that the base grease with graphite did not represent better lubricating performance than that with MoS_2_, but it largely improved in tribological quality relative to the base grease. C_60_ itself is decidedly inferior in lubricating performance as compared with the generally used lubricating additives such as MoS_2_ and graphite. Here, it should be noted that the WSD and wear volume loss of the nanocomposite materials were 0.41 mm and 0.5 × 10^−3^ mm^3^, respectively. Thus it is found that the nanocomposite prepared shows a much better lubricating performance than that with other additives. This result suggests that a C_60_ monolayer between graphenes strongly influences lubricating performance. Further detailed investigations of the tribological behaviors and the lubrication mechanism of the nanocomposite are now in progress.

## 4. Conclusions

At present, preliminary experiments indicate that it is possible to intercalate huge fullerene molecules such as C_70_ and La@C_82_, larger than a C_60_ molecule, into graphite. Up until now, we have studied C_60_ monolayers confined by graphite flakes [7] and C_60_ monolayers included among graphite [8,9,10], which, interestingly, exhibits ultralow friction, because C_60_ molecules act as molecular bearings. We investigated lubricating properties of nanocomposite materials in commercial grease. The grease with the nanocomposite materials provides a much better lubricating performance than that with other additives that have been well-known up to now. This result exhibits that a C_60_ monolayer intercalated between graphenes plays an important role for the lubricating behaviors.
